# Decreased cardiac output: an integrative review

**DOI:** 10.1590/0034-7167-2022-0265

**Published:** 2023-02-06

**Authors:** Ricardo Costa da Silva, Micaelle Costa Gondim, Gabriela Moreira Melo, Viviane Martins da Silva, Agueda Maria Ruiz Zimmer Cavalcante, Miriam de Abreu Almeida, Amália de Fátima Lucena

**Affiliations:** I Universidade Federal de Goiás. Goiânia, Goiás, Brazil; II Universidade Federal do Ceará. Fortaleza, Ceará, Brazil; III Universidade Federal do Rio Grande do Sul. Porto Alegre, Rio Grande do Sul, Brazil

**Keywords:** Cardiac Output, Nursing Diagnosis, Review, Evidence-Based Nursing, Standardized Nursing Terminology, Gasto Cardíaco, Diagnóstico de Enfermería, Revisión, Enfermería Basada em la Evidencia, Terminología Normalizada de Enfermería, Débito Cardíaco, Diagnóstico de Enfermagem, Revisão, Enfermagem Baseada em Evidência, Terminologia Padronizada em Enfermagem

## Abstract

**Objective:**

to identify, in the scientific literature, the defining characteristics and contributing factors (related factors, associated conditions and populations at risk) for nursing diagnosis decreased cardiac output.

**Method:**

an integrative literature review, conducted between September and October 2020, with an update in March 2022, in the MEDLINE via PubMed, LILACS, SciELO, CINAHL and EMBASE databases. Using acronym PEO, studies published in the last 10 years in Portuguese, English and Spanish were included. A descriptive analysis was carried out to present the elements mapped in the literature.

**Results:**

analysis of 31 articles identified different elements, highlighting 4 new related factors: hyperglycemic stress, prone position, left lateral position, sleep deprivation. Individuals with a history of cardiovascular disease and males were identified as possible populations at risk.

**Final considerations:**

the elements for decreased cardiac output, identified in the literature, add evidence that justifies the permanence of this diagnosis in the NANDA-I classification.

## RESUMO

**Objetivo:**

identificar, na literatura científica, as características definidoras e fatores contribuintes (fatores relacionados, condições associadas e populações em risco) para o diagnóstico de enfermagem débito cardíaco diminuído.

**Método:**

revisão integrativa da literatura, conduzida entre setembro e outubro de 2020, com atualização em março de 2022, nas bases de dados MEDLINE via PubMed, LILACS, SciELO, CINAHL e EMBASE. Com uso do acrônimo PEO, foram incluídos estudos publicados nos últimos 10 anos em português, inglês e espanhol. Realizouse análise descritiva para apresentar os elementos mapeados na literatura.

**Resultados:**

análise de 31 artigos identificou diferentes elementos, com destaque para 4 novos fatores relacionados: estresse hiperglicêmico, posição prona, posição lateral esquerda, privação do sono. Indivíduos com história de doença cardiovascular e do sexo masculino foram apontados como possíveis populações em risco.

**Considerações finais:**

os elementos para débito cardíaco diminuído, identificados na literatura, agregam evidências que justificam a permanência desse diagnóstico na classificação da NANDA-I.

## RESUMEN

**Objetivo:**

identificar, en la literatura científica, las características definidoras y los factores contribuyentes (factores relacionados, condiciones asociadas y poblaciones de riesgo) para el diagnóstico de enfermería de gasto cardíaco disminuido.

**Método:**

revisión integrativa de la literatura, realizada entre septiembrey octubre de 2020, con actualización en marzo de 2022, en las bases de datos MEDLINE vía PubMed, LILACS, SciELO, CINAHL y EMBASE. Utilizando la sigla PEO, se incluyeron estudios publicados en los últimos 10 años en portugués, inglés y español. Se realizó un análisis descriptivo para presentar los elementos mapeados en la literatura.

**Resultados:**

el análisis de 31 artículos identificó diferentes elementos, con énfasis en 4 nuevos factores relacionados: estrés hiperglucémico, posición prona, posición lateral izquierda, privación del sueño. Las personas con antecedentes de enfermedad cardiovascular y los hombres se identificaron como posibles poblaciones en riesgo.

**Consideraciones finales:**

los elementos para gasto cardíaco disminuido, identificados en la literatura, suman evidencias que justifican la permanencia de este diagnóstico en la clasificación NANDA-I.

## INTRODUCTION

Standardized language systems in nursing organize the vocabulary scope of concepts and elements related to the phenomena that nurses must identify, treat and assess in health care^(1)^. In the clinical setting, changes in the capacity of the blood volume required for circulation, called cardiac output^(2)^, can produce signs and symptoms resulting from hypoperfusion that are of special interest to nursing.

Decreased cardiac output (DCO) (00029) is a nursing diagnosis (ND) from NANDA International, Inc. (NANDA-I)^(3)^, defined as “an inadequate volume of blood pumped by the heart to meet the metabolic demands of the body”. It is contained in the Activity/Rest domain and has 36 defining characteristics (DC), divided into five groups: altered heart rate/rhythm, altered preload, altered afterload, altered contractility, behavioral/emotional^(3)^. By DC, we understand the set of observable clues or inferences that are grouped as manifestations of an ND. As for associated conditions (conditions not modifiable by a nurse), six are described for DCO: altered contractility, altered heart rate, altered afterload, altered preload, altered heart rhythm and altered stroke volume^(3)^.

Although DCO has been present in the classification since 1975 and has already been the focus of conceptual research regarding its pertinence in the field of nursing^(4)^, to date, previous studies have not identified antecedent elements that show a causal relationship with this human response, called related factors (RF), and that are subject to modification by independent nursing interventions^(3)^. It is also observed that, although DCO is often identified in people with cardiovascular diseases, especially heart failure^(5)^, its diagnostic structure does not include a description of populations at risk, which are defined as a group of people who share common characteristics and who, due to such characteristics, are more susceptible to certain human responses^(3)^.

NANDA-I^(3)^ is the only classification that presents well-defined criteria regarding validity evidence levels of ND present in its structure, defined so far as level of evidence (LoE). Therefore, each ND must present a set of evidence (theoretical and clinical) that allow its correct interpretation, from a set of manifestations for certain clinical contexts^(3)^. In this context, DCO does not present the minimum level of evidence required by NANDA-I to justify its permanence in the classification, and its withdrawal is suggested in the next edition 2024-2026.

Therefore, DCO refinement from the literature allows identifying possible contributing factors (RF, associated conditions, populations at risk) that explain the causal dynamics of this ND as well as the characterization of its occurrence in the scenarios in which it has been identified. In addition to aggregating the evidence necessary for DCO to remain in NANDA-I classification, such information will assist in the clinical reasoning and decision-making of nurses, providing evidence that explains the cause-effect relationships of this ND, which may reflect a greater degree of accuracy in the process of identifying this human response in the clinical context.

## OBJECTIVE

To identify, in scientific literature, the DC and contributing factors (RF, associated conditions, and populations at risk) for ND DCO.

## METHODS

### Ethical aspects

This study was carried out in public domain databases, which does not require submission to a Research Ethics Committee.

### Study design

This is an integrative literature review^(6)^, developed in six stages: theme identification; guiding question selection; inclusion and exclusion criteria establishment; definition of the information to be extracted from selected studies; assessment of included studies; and interpretation of results.

### Study protocol

To meet the purposes of this review, the following guiding question was used: what are the DC resulting from DCO and what are the possible contributing factors (RF, associated conditions and populations at risk) for the occurrence of this phenomenon? The formulation of the question considered an adaptation of acronym PEO^(7)^, as: P (Population of interest) = general population; E (Exposure of interest) = DC, RF, associated conditions and populations at risk; and O (Outcome) = DCO occurrence.

Data collection took place between September and October 2020, with an update in March 2022, with searches in the electronic databases: MEDLINE via PubMed, Latin American and Caribbean Literature in Health Sciences (LILACS), Cumulative Indexto Nursing and Allied Health Literature (CINAHL), EMBASE and SciELO. The Descriptors in Health Sciences (DeCS), Medical Subject Heading (MeSH), CINAHL and Emtree titles for EMBASE were used. The search strategy followed the criteria of each database with Boolean operators AND and OR, crossed between controlled and uncontrolled descriptors, followed by NOT to exclude studies related to the risk of DCO.

In each base, two strategies were used for a more detailed understanding of the phenomenon of interest^(6)^. In the broad strategy, the objective was to rescue studies that presented possible new elements that precede (contributing factors) or that are a consequence (DC) of the presence of the DCO phenomenon, without the use of descriptors related to nursing. In the restricted strategy, the objective was to rescue studies directly related to DCO, in order to verify how this diagnosis has been presented in nursing and in which contexts and populations (Chart 1). Moreover, the references present in the articles identified and selected by the search strategy were also consulted.

**Chart 1:** Search strategy used in the databases, Brazil, 2022

	Controlled descriptors	Uncontrolled descriptors
**Strategy 1 (broad)**	Cardiac Output [MeSH Terms]; Cardiac Output, Low [MeSH Terms]; cardiac output, decreased [Título CINAHAL];‘heart output’ [emtree]; Débito Cardíaco [DeCS]; Baixo Débito Cardíaco [DeCS]	decreased; risk; diminuído; risco
**Strategy 2 (restricted)**	Cardiac Output [MeSH Terms]; Cardiac Output, Low [MeSH Terms]; cardiac output, decreased [Título CINAHAL]; Débito Cardíaco [DeCS]; Baixo Débito Cardíaco [DeCS]; ‘heart output’ [emtree]; NANDA Nursing Diagnoses [Título CINAHAL];‘nursing diagnosis’ [emtree]	decreased; risk; diminuído; risco; nursing diagnos*; Diagnósticos de Enfermagem

Studies published in the last 10 years were included, without restriction of sample size, in English, Portuguese and Spanish. The time frame is justified by the volume of articles retrieved not related to the scope of this review, such as articles on validation of techniques and/or equipment for measuring cardiac output at the bedside, in addition to experimental studies with animals. Review studies, case studies, textbooks, editorials, protocols, diagnostic performance studies, studies that did not directly describe DCO and studies in which there was no significant and/or explained reduction in cardiac output were also excluded.

Eligible studies were retrieved on the same day to avoid bias as the databases are updated daily. To eliminate possible duplicate articles and speed up the initial screening of the data, the results were loaded into Rayyan® software, which uses a reliable semi-automation process, incorporating a high level of usability and efficiency into the analysis process^(8)^. The results were analyzed blindly and independently by two researchers, graduate students with research in the field of cardiology and nursing diagnoses, and disagreements were resolved by consensus with a third researcher.

**Figure 1:**
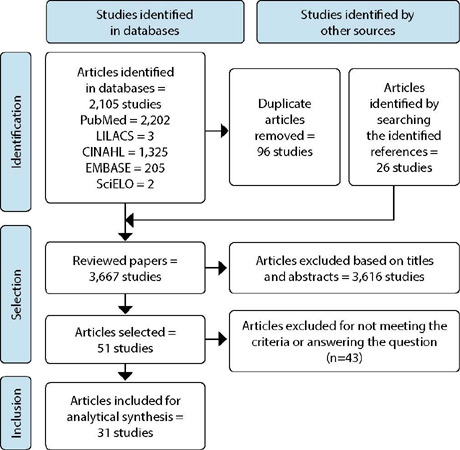
Flowchart of the stages of search and selection of analyzed articles, Brazil, 2022

Finally, 51 studies were selected for full reading, of which 31 made up the final sample, as shown in Figure 1, following the Preferred Reporting Items for Systematic reviews and Meta-Analyses (PRISMA) recommendations^(9)^.

### Data analysis

The information from the selected articles was organized in a Microsoft Office Excel spreadsheet (2019), containing the essential items of each study: bibliographic data, study objectives, methodological design, sample size and characteristics, DC and possible contributing factors to DCO.

The cross-mapping technique^(10)^ was applied between the terms found in the literature and the elements contained in the NANDA-I for DCO, in order to look for possible similarities. In this process, the following steps were adopted: a) verify semantic equivalence between terms in the literature and terms in NANDA-I for DCO; b) verify the conceptual equivalence between terms in the literature and terms in NANDA-I for DCO; c) highlight terms not matching DCO as possible new DC and contributing factors; d) verify in NANDA-I whether the possible new terms are already described for other diagnoses, considering the standardization of terms in taxonomic structure.

Furthermore, the publications were qualified according to the level of scientific evidence proposed by Fineout-Overholt^(11)^, considering the criteria adopted in this study: level II - evidence derived from at least one well-designed randomized controlled clinical trial; level III - evidence obtained from well-designed clinical trials without randomization; level IV - evidence from well-designed cohort and case-control studies; level VI - evidence derived from a single descriptive or qualitative study.

Data analysis and presentation were performed descriptively. The results were synthesized and grouped in synoptic tables, distributed from the DC and the possible contributing factors to DCO, coded in chronological order (S1, S2,…).

## RESULTS

Chart 2 presents a summary of the characterization process and assessment of the level of evidence of selected studies.

**Chart 2:** Characterization of selected studies using the selection flowchart (N=31), Brazil, 2022

Study	Author, year, and country	Design	Objective	LoE	Context	DCO
S1^(12)^	Garan et al., (2010) - USA	Retrospective longitudinal	Investigate whether peripheral vascular compensatory mechanisms are preserved after orthotopic heart transplantation.	IV	36 medical records of hospitalized patients in 38 months	Phenomenon
S2^(13)^	Paganin et al., (2010) - Brazil	Cross-sectional	Map the Nursing Interventions Classification interventions with the most prevalent NDs in the first 24 hours of patient admission to the Intensive Care Unit.	VI	150 medical records of hospitalized individuals	ND
S3^(14)^	Paganin et al., (2010) - Brazil	Cross-sectional	Identify the main ND established in an Intensive Care Unit and compare them between clinical and surgical patients.	VI	150 medical records of hospitalized individuals	ND
S4^(15)^	Martins, Aliti, Rabelo (2010) - Brazil	Cross-sectional	Validate DCO in patients with decompensated HF hospitalized in Intensive and Emergency Care Units.	VI	29 hospitalized patients	ND
S5^(16)^	Alessandra, Silla, Marilisia (2011) - Italy	Cross-sectional	Describe the use of nursing terminology in care plans provided to patients in a cardiology rehabilitation unit.	VI	76 outpatient patients	ND
S6^(17)^	Aliti etal., (2011) - Brazil	Cross-sectional	Identify the signs and symptoms to infer the main ND in patients with decompensated HF.	VI	303 hospitalized patients	ND
S7^(18)^	Bodetofta et al., (2011) - Sweden	Randomized clinical trial	Check whether inhaling oxygen decreases cardiac output and blood flow.	II	16 healthy volunteers	Phenomenon
S8^(19)^	Pereira et al., (2011) - Brazil	Cross-sectional	Identify the frequency of ND and DC in patients with cardiovascular diseases and characterize them in terms of sociodemographic and clinical variables.	VI	30 hospitalized patients	ND
S9^(20)^	Scherb et al., (2011) - USA	Multicentric comparative	Compare the ten most frequent diagnoses, interventions and nursing outcomes in patients hospitalized for HF.	VI	302 medical records of hospitalized patients	ND
S10^(21)^	Matos et al., (2012) - Brazil	Cross-sectional	Assess the prevalence of DC and to analyze which are the predictive factors of DCO in patients with HF undergoing assessment for heart transplantation.	VI	38 hospitalized patients using Pulmonary Artery Catheter	ND
S11^(22)^	Kyhl et al., (2013) - Denmark	Non-randomized clinical study	Check whether positive pressure ventilation reduces cardiac output.	III	18 healthy volunteers	Phenomenon
S12^(23)^	Wajima et al., (2013) - Japan	Randomized clinical trial	Identify and compare hemodynamic parameters for probable hypotension during patient commuting to bed after different types of anesthesia (general, epidural or spinal combined with general).	II	69 patients hospitalized in the immediate postoperative period of elective surgery	Phenomenon
S13^(24)^	Hargens et al., (2015) - USA	Randomized clinical trial	Determine whether resting hemodynamic variables are altered in individuals with OSA entering cardiac rehabilitation compared to those without apnea.	II	73 individuals in outpatient cardiac rehabilitation	Phenomenon
S14^(25)^	Kasai et al., (2015) - Canada	Non-randomized clinical trial	Investigate whether, in patients with HF, those with OSA have greater reductions in stroke volume and cardiac output and whether these decreases are proportional to the severity of OSA.	III	60 outpatient patients	Phenomenon
S15^(26)^	Li et al., (2015) - China	Case-control study	Investigate total peripheral vascular resistance, cardiac output and natriuretic peptide levels in children with PTS during supine, orthostatic and return to supine positions.	IV	29 children with PTS in the case group and 32 controls	Phenomenon
S16^(27)^	Ma et al., (2015) - USA	Longitudinal	Assess the short-term cardiovascular response to prone position in neonates in neonatal intensive care.	IV	30 neonates hospitalized for 10 minutes	Phenomenon
S17^(28)^	Souza et al., (2015) - Brazil	Cross-sectional	Assess the clinical usefulness of operational definitions for DC DCO, excessive fluid volume and impaired activity tolerance, and the concomitant presence of these diagnoses in patients with HF decompensation.	VI	25 hospitalized patients	ND
S18^(29)^	Chikhani et al., (2016) - England	Non-randomized clinical study	Investigate the effect of prone position using surgical pads on hepatic blood flow and cardiovascular behavior in healthy subjects.	III	10 healthy volunteers	Phenomenon
519^(30)^	Costa, Linch, Souza (2016) - Brazil	Cross-sectional	Identify the main signs and symptoms of patients with heart disease admitted to an intensive cardiac care unit and infer the main ND.	VI	77 medical records of hospitalized individuals	ND
S20^(31)^	Galvão et al., (2016) - Brazil	Cross-sectional	Identify priority NDs for patients with decompensated HF admitted to a cardiology emergency room at a university hospital.	VI	62 hospitalized patients	ND
S21^(32)^	Pereira et al., (2016) -Brazil	Longitudinal	Identify the ND fatigue, DCO and activity intolerance and verify the association of DD with the respective ND in patients hospitalized with HF.	IV	72 patients followed up during three weeks of hospitalization	ND
S22^(33)^	Sánchez et al., (2016) - Colombia	Cross-sectional	Determine the clinical and construct validity of ND DCO in patients with HF.	VI	200 hospitalized patients	ND
S23^(34)^	Yang et aI., (2016) - China	Randomized clinical trial	Explore the effects of stress hyperglycemia on cardiac function and prognosis in critically ill intensive care patients.	II	80 hospitalized patients	Phenomenon
S24^(35)^	Miró et al., (2017) - Italy	Non-randomized clinical study	Investigate changes in left ventricular systolic function due to thoracic epidural anesthesia and changes in hemodynamic variables and left ventricular diastolic function in patients undergoing major abdominal surgery.	III	24 hospitalized without significant heart disease	Phenomenon
S25^(36)^	Paviotti, Todero, Demarini (2017) - Italy	Longitudinal	Verify the short-term effects of the left lateral position on the cardiovascular parameters of hemodynamically stable newborns.	IV	32 neonates hospitalized for 10 minutes	Phenomenon
S26^(37)^	Nederend et al., (2018) - Netherlands	Randomized clinical trial	Assess cardiac autonomic nervous system activity and cardiac function in children after aortic coarctation repair and investigate the relationship between the two.	II	31 outpatient patients	Phenomenon
S27^(38)^	Slomko et al., (2018) - Poland	Randomized clinical trial	Analyze the impact of sleep deprivation on hemodynamic and autonomic parameters in subjects with normal blood pressure, compared with prehypertension and hypertension, at 24, 28 and 32 hours of total sleep deprivation.	II	30 healthy volunteers	Phenomenon
S28^(39)^	Stewart et al., (2018) - USA	Case-control study	Investigate whether postural hyperventilation is one of the causes of PTS and whether there is associated hyperventilation when there is a reduction in cardiac output.	IV	58 cases with PTS and 16 healthy controls	Phenomenon
S29^(40)^	Ribeiro et al., (2019) - Brazil	Cross-sectional	Assess ND Frail Elderly Syndrome in older adults with chronic diseases in a health district in the Federal District.	VI	78 community older adults	ND
S30^(41)^	Zhang et al., (2021) - China	Case-control study	Analyze the associations of cardiac function with inflammatory cytokines, oxidative stress and anemia in patients with uremia.	IV	43 patients in uremia in the case group and 36 patients in the control group	Phenomenon
S31^(42)^	Perry et al.,(2021)- England	Longitudinal	Describe hemodynamic differences in women with gestational hypertension.	IV	717 pregnant women followed up during 77 months	Phenomenon

*S - study; LoE - level of evidence; ND - Nursing Diagnosis(s); HF - heart failure; DCO - Decreased Cardiac Output; OSA - obstructive sleep apnea; PTS - Postural Tachycardia Syndrome.*

Of the 31 studies analyzed in the review, 17 (55%)^(12, 18, 22-27, 29, 34-39, 41)^ referred to the DCO phenomenon, and 14 (45%)^(13-17, 19-21, 28, 30-33,40)^, to DCO. The studies were mostly carried out in countries in South America (n=12;38%)^(13-15, 17, 19, 21, 28, 30-33, 40)^ and in Europe (n=9;29%)^(16, 18, 22, 29, 35-38, 42)^, with emphasis on Brazilian publications (n=11; 35%)^(13-15, 17, 19, 21, 28, 30-32, 40)^, that were exclusively related to DCO. In publications related to ND, which aimed to identify its occurrence in an isolated way (n=3;21%)^(15, 21, 33)^ or together with other diagnoses (n=i11.79%)^(13-14, 16-17, 19-20, 28, 30-32, 40)^, the breadth in the prevalence estimates is highlighted, which ranged from 6.7%^(13-14)^ to 100%^(15-16, 28, 33)^.

Despite the heterogeneity of the population profiles and methodological designs, the studies showed similarities that they were carried out with samples of people with cardiovascular disorders, such as heart failure (HF) (n=11;35%)^(15, 17, 19-21, 25, 28, 31-33, 40)^, hypertension (n=7;22%)^(16, 19, 28-29, 38, 40, 42)^ or with a history of acute myocardial infarction (n=4; 13%)^(16, 24, 30, 40)^. In addition, males were highlighted as having the highest composition among the samples (n=10;32%)^(14-15, 17, 24-25, 28, 31-33, 35)^. As for the level of evidence, level VI was the most frequent (n=13;42%)^(13-17, 19-21, 28, 30-31, 33, 40)^, followed by level IV studies (n=8;26%)^(12, 26-27, 32, 36, 39, 41-42)^.

Regarding the studies that presented the DC currently described in NANDA-I for DCO, *edema* ^(13, 15, 17, 19, 21, 28, 30, 32-33, 40)^ was the most frequent in the literature (n=10;71%) *Jugular vein distension* ^(15, 17, 19, 28, 32-33)^, dyspnea^(15, 17, 19, 30, 32-33)^ and *fatigue* ^(13, 15, 19, 28, 32-33)^ were present in six publications (43%) each. Other studies have pointed out *abnormal skin color* ^(13, 15, 28, 32-33)^ and *decreased ejection fraction* ^(17, 21, 28, 32-33)^(n=5; 36%), in addition to *clammy skin* ^(13, 15, 28, 33)^ and *presence of S3 heart sound* ^(21, 28, 32-33)^ (n=4;29%). *Bradycardia* ^(21, 30, 33)^, *nocturnal paroxysmal dyspnea* ^(15, 17, 33)^ *heart palpitations* ^(15, 33, 40)^ *altered blood pressure* ^(28, 33, 40)^ *orthopnea* ^(15, 17, 33)^, *tachycardia* ^(21, 30, 33)^, *prolonged capillary refill* ^(13, 28, 33)^ and *cough* ^(15, 28, 33)^were presented in three studies (21%) each.

Other DC also described in NANDA-I for DCO were cited with lower prevalence. *Electrocardiogram change* ^(13, 28)^, *psychomotor agitation* ^(28, 30)^, *anxiety* ^(28, 30)^, *weight gain* ^(15, 33)^, *decreased peripheral pulses* ^(28, 33)^, *increased central venous pressure* ^(15, 21)^, *presence of S4 heart sound* ^(28, 33)^, *adventitious breath sounds* ^(15, 33)^ and *oliguria* ^(15, 28)^ were present in two studies (14%) each. *Increased pulmonary artery occlusion pressure* ^(21)^, *decreased central venous pressure* ^(21)^, *increased pulmonary vascular resistance* ^(21)^, *decreased pulmonary vascular resistance* ^(21)^ and *increased systemic vascular resistance* ^(21)^ were cited in one study (7%) each.

The following DC of DCO present in NANDA-I were not found in this review: *decreased pulmonary artery occlusion pressure, heart murmur, decreased systemic vascular resistance, decreased cardiac index, decreased stroke volume index, and decreased left ventricular stroke work index.* Moreover, some of the DC that were identified in the literature are not present in the structure of DCO, although they are part of other NANDA-I diagnoses, such as *arrhythmias* ^(13, 28, 32-33)^, *ascites* ^(28)^, *hepatomegaly* ^(15)^, *restlessness* ^(13)^, *positive hepatojugular reflex* ^(28)^, *altered mental status* ^(28)^ and *altered breathing pattern* ^(28)^. No new DC were observed other than those already present in the classification.

Regarding associated conditions currently described in NANDA-I for DCO, *altered contractility* ^(13, 31)^, *altered heart rate* ^(13, 31)^ and *altered stroke volume* ^(13, 31)^ were identified in two studies (14%). Still, *altered heart rate* ^(31)^ and *altered postload* ^(13)^ were also cited in one study (7%) each. Finally, the contributing factors (RF, associated conditions and populations at risk) identified in the literature and that did not correspond to DCO in NANDA-I are grouped in Table 1.

**Table 1:** Possible new contributing factors for diagnosis decreased cardiac output, Brazil, 2022

Possible new related factors	Studies
Hyperglycemic stress	S23^(34)^
Prone position	S16^(27)^;S18^(29)^
Left side position	S25^(36)^
Sleep deprivation	S27^(38)^

Possible new associated conditions	
Obstructive sleep apnea	S13^(24)^; S14^(25)^
Coarctation of the aorta	S26^(37)^
Gestational hypertension	S31^(42)^
Anesthetic procedure	S24^(35)^
Postural tachycardia syndrome	S15^(26)^; S28^(39)^
Transplantation	S1^(12)^
Oxygen therapy	S7^(18)^
Uremia	S30^(41)^
Positive pressure ventilation	S11^(22)^

Possible new populations at risk	
Individuals with a history of cardiovascular disease	S4^(15)^; S5^(16)^; S6^(17)^; S8^(19)^; S9^(20)^; S10^(21)^; S13^(24)^; S14^(25)^; S17^(28)^; S19^(30)^; S20^(31)^; S21^(32)^; S22^(33)^; S27^(38)^; S29^(38)^; S31^(42)^
Male	S3^(14)^; S4^(15)^; S6^(17)^; S13^(24)^; S14^(25)^; S17^(28)^; S20^(31)^; S21^(32)^; S22^(33)^; S24^(35)^

## DISCUSSION

The results of this review allowed the answer to the proposed guiding question, corroborating the main DC representative of DCO already described in NANDA-I^(3)^, as well as the identification of possible new contributing factors to this ND, with emphasis on four RF that can be independently modified by a nurse: *hyperglycemic stress* ^(34)^, *prone position* ^(27, 29)^, *left side position* ^(36)^ *and sleep deprivation* ^(38)^.

The highest prevalence of studies in this review was those conducted in South American countries, which demonstrate their potential for research, despite being developing countries with limitations in the process of translation of evidence^(43)^. This reinforces what is advocated by NANDA-I as an international entity regarding the need for studies on ND in different care contexts, in order to generate evidence for clinical and population validation^(44)^.

Despite previous questions about the independence of DCO as a specific and non-collaborative phenomenon with other subjects^(4, 5)^, the high frequency of studies in nursing^(13-17, 19-21, 28, 30-33, 40)^ suggests that there is a lot of interest in this phenomenon, since it has been described in different practice scenarios, with potential for interventions and positive health outcomes from the specific management of nursing.

Regarding the level of evidence of specific investigations on DCO, the studies that presented DC were mostly cross-sectional and focused on the prevalence of this diagnosis. Although crucial for clinical and social validity in specific subgroups, advances are needed in conducting diagnostic studies focusing on the predictive and prognostic capacity of these DC for further submission to NANDA-I^(3, 45)^.

The group of DC already described in NANDA-I^(3)^, as consequences of changes in preload, were the most frequent, especially *edema, fatigue* and *jugular vein distension. Edema* is a sign of HF, as it represents the deficit of the right ventricle in ejecting the entire amount of blood from the venous circulation, leading to increased hydrostatic pressure in the organs and capillaries^(2)^. *Fatigue* results from inadequate distribution of oxygenated volume by reduced output and inadequate excretion of the products of metabolism. *Jugular vein distension* reflects the increases in pressure and volume in the right atrium^(2, 46)^.

Regarding contractility, *decreased ejection fraction* is a result of the amount of blood available in the left ventricle between systole and diastole, being an important characteristic for the classification of HF^(47)^ On the other hand, *nocturnal paroxysmal dyspnea* occurs due to the addition of daytime fluids that, at night, can return to the blood circulation, causing accumulation in the pulmonary alveoli, reduced gas exchange and increased carbon dioxide, with consequent awakening by patients^(2, 46)^.

In the altered postload group, *dyspnea* and *abnormal skin color* stood out. *Dyspnea* can occur with minimal or moderate activities, being an important predictor of HF severity^(46)^, indispensable for clinical nursing care to people with cardiovascular alterations. In addition to this, *abnormal skin color* represented by cyaniasis or skin pallor are initial signs of tissue hypoperfusion^(48)^.

Altered heart rate/rhythm was identified by *bradycardia, tachycardia* and *heart palpitations. Bradycardia* appears as a result of exhaustion of the heart muscle in generating output. As a consequence, reduced stroke volume can activate the sympathetic nervous system and increase heart rate, generating *tachycardia* and *heart palpitations* ^(2, 46)^. It is emphasized that DC *electrocardiogram change* does not describe which alterations should be considered. The reformulation of this characteristic would be relevant for its better understanding and identification in clinical practice.

Finally, behavioral alterations were the least identified in the literature, with *anxiety* and *psychomotor agitation.* Both can be identified in a more severe spectrum of DCO, since low output alters cerebral perfusion, creating a vicious cycle of worsening concomitantly with *dyspnea* ^(2, 46)^.

In general, the evidence about the DC extracted from the literature supports the permanence of DCO in NANDA-I classification^(3)^ as a phenomenon identified and researched in different scenarios, adding the minimum necessary evidence. However, when considering the current structure presented in the classification, in which DC are grouped into subleveis and named by components similar to those presented in the associated conditions, this can make it difficult to understand their elements. A restructuring, for example, at different levels of severity, could facilitate the explanation and relationship between diagnostic components.

Consistently, the most frequent DC for DCO indicated in the literature are initial changes in debt that are not restricted to intensive care environments, justifying their applicability to different clinical contexts. For its correct identification, DCO requires a solid training of nurses, which requires transversality of knowledge, which translates into skills for the physical examination, ability to use and interpret diagnostic support technologies and analysis of functional patterns for the applicability of knowledge to nursing.

Although NANDA-I presents DC obtained by direct measurement of DC, which can be identified from the presence of a pulmonary artery catheter installed in individuals (considered the gold standard), advances in clinical and interventional cardiology have prioritized non-invasive or minimally invasive measurement, with a focus on patient safety^(46, 48)^.

The installation of invasive hemodynamic monitoring measures depends on the indication by the medical team beyond patients’ clinical spectrum of severity. These may be justifications for the low frequency or even absence of some of the DC that correspond to measurements necessarily obtained by a pulmonary artery catheter. The non-observation of these signs in the literature points to the need for reformulation or inclusion of new parameters obtained by minimally invasive measures, since they are not being identified in clinical practice, as previously observed in a systematic review of the DC of DCO^(5)^. Furthermore, in the study conducted by Matos et al.^(21)^, *increased pulmonary artery occlusion pressure, increased central venous pressure, decreased central venous pressure, and decreased pulmonary vascular resistance* did not increase the chances of diagnosis occurring.

There are some DC rescued in the literature that are already present in NANDA-I classification^(3)^, but described in other diagnoses, such as excessive fluid volume. Such signs and symptoms are derived from cardiac congestion due to pump failure and reduced output, with consequent accumulation in body fluid volume, as is the case with HF^(47)^. For this situation, studies of differential analysis can contribute to the identification of DC specific to correlated phenomena^(45)^. In the case of DC *arrhythmias,* this appears to be a granularity of *electrocardiogram change.* Studies that develop conceptual and operational definitions are especially useful to help with such impasses.

The studies that identified possible RF or associated conditions are the result of methodological designs with a higher level of evidence, which allow the attribution of cause and effect, representing the second highest frequency found. Studies with greater methodological robustness are needed to add validity of specific causes to DCO, being encouraged to better clarify the relationships between its antecedent elements.

Regarding the possible RF, the presence of *hyperglycemic stress* ^(34)^, *prone position* ^(27, 29)^, *left lateral position* ^(36)^ and *sleep deprivation* stand out^(38)^ as subject to independent interventions by nurses, with nursing outcomes and interventions available in the Nursing Outcomes Classification (NOC)^(49)^ and Nursing Interventions Classification (NIC)^(50)^.

Patients who present hyperglycemic stress, in intensive care, secondary to trauma, infections, major surgeries, among other causes, present significant changes in hemodynamic parameters, such as reduced cardiac output, as a result of worsening cardiac function. This fact may be due to the reduction in muscle strength and myocardial energy capacity after failure in compensatory mechanisms^(34)^. Interventions for hyperglycemia management (2120) and nutritional therapy (1120) can manage these cases^(50)^.

The reduction in cardiac output from positioning patients in *prone position* ^(27, 29)^ or *left lateral position* ^(36)^ is a direct result of the increase in intrathoracic pressure, which generates a reduction in venous return and consequent reduction in systolic volume^(27, 29, 36)^. Nursing activities related to positioning (0840) can avoid such complications^(50)^.

Periods of *sleep deprivation can* increase the dominance of the sympathetic nervous system in the body, linking pathophysiological mechanisms of blood pressure control centers, with a consequent reduction in cardiac output^(38)^. Both sleep disorder and sleep deprivation are ND described in NANDA-I that are common in the hospital environment^(3)^. Sleep enhancement (1850) may contribute to the resolution of this agent^(50)^.

Associated conditions already available in NANDA-I and which are related to DCO were rarely mentioned in the included studies, in addition to *altered preload,* which was not verified in the literature, which confirms the need to review this diagnostic element. The addition of associated conditions identified from this literature review can improve the clinical reasoning of nurses in clinical practice, with the investigation of initial signs and symptoms in specific contexts., with emphasis on *obstructive sleep apnea* ^(24-25)^ and *postural tachycardia syndrome* ^(26, 39)^, which contribute to observable changes in nursing assessment.

Finally, according to the findings of this review, individuals with a history of cardiovascular disease and *males* are likely to be populations at risk for DCO, sharing common characteristics related to heart pump failure, in addition to being frequent demographic profiles in the clinical investigation contexts observed in this review.

### Study limitations

The limitation of time for the search for publications, as well as the search strategies used, are a characteristic limitation of this study design, since there may be other publications outside the stipulated period.

### Contributions to nursing

The deepening of DCO knowledge, based on scientific evidence that supports these phenomena, allows the proposal to refine this ND in NANDA-I classification, promoting the strengthening of this diagnostic classification and the advancement of nursing knowledge. In addition, it contributes to better decision-making in the search for better health outcomes, which affects the qualification of clinical practice.

## FINAL CONSIDERATIONS

This study made it possible to identify DC and contributing factors for the DCO present in the literature, adding evidence that justifies its permanence in NANDA-I classification, especially by verifying causal factors that are subject to intervention and independent treatment by nursing.

As it is a complex phenomenon, future investigations on the diagnostic concept are necessary for a better understanding of its essential attributes, with a view to solving possible impasses regarding its definition and its constitutive elements that help its identification by practice nurses. It is suggested that, based on these results, new studies of clinical-causal validation and clinical construct are carried out, to strengthen evidence about DCO with higher levels of evidence, especially in non-intensive care settings.
